# The Relationship between Environmental Dioxygen and Iron-Sulfur Proteins Explored at the Genome Level

**DOI:** 10.1371/journal.pone.0171279

**Published:** 2017-01-30

**Authors:** Claudia Andreini, Antonio Rosato, Lucia Banci

**Affiliations:** 1 Magnetic Resonance Center, University of Florence, Sesto Fiorentino, Italy; 2 Department of Chemistry, University of Florence, Sesto Fiorentino, Italy; CINVESTAV-IPN, MEXICO

## Abstract

About 2 billion years ago, the atmosphere of the Earth experienced a great change due to the buildup of dioxygen produced by photosynthetic organisms. This transition caused a reduction of iron bioavailability and at the same time exposed living organisms to the threat of oxidative stress. Iron-sulfur (Fe-S) clusters require iron ions for their biosynthesis and are labile if exposed to reactive oxygen species. To assess how the above transition influenced the usage of Fe-S clusters by organisms, we compared the distribution of the Fe-S proteins encoded by the genomes of more than 400 prokaryotic organisms as a function of their dioxygen requirements. Aerobic organisms use less Fe-S proteins than the majority of anaerobic organisms with a similar genome size. Furthermore, aerobes have evolved specific Fe-S proteins that bind the less iron-demanding and more chemically stable Fe_2_S_2_ clusters while reducing the number of Fe_4_S_4_-binding proteins in their genomes. However, there is a shared core of Fe-S protein families composed mainly by Fe_4_S_4_-binding proteins. Members of these families are present also in humans. The distribution of human Fe-S proteins within cell compartments shows that mitochondrial proteins are inherited from prokaryotic proteins of aerobes, whereas nuclear and cytoplasmic Fe-S proteins are inherited from anaerobic organisms.

## Introduction

During the first billion years of life on the Earth, the environment was anaerobic. Iron and sulfur were abundant, and they were used for the formation of iron-sulfur (Fe-S) clusters as cofactors of proteins. Fe-S clusters contain two or more iron ions bridged by sulfide ions. Each iron ion is tetracoordinated, with its coordination sphere typically completed by the sulfur or nitrogen atoms of cysteine and histidine side chains, respectively [1–3]. Often, the metal site of rubredoxin, which contains a single iron ion coordinated by four cysteines, is regarded as the simplest unit of Fe-S clusters. Fe-S clusters are among the most versatile inorganic cofactors [4]. They are involved in a plethora of functional processes, including aerobic as well as anaerobic respiration, regulation of gene expression, amino acid and nucleotide metabolism, DNA modification and repair and tRNA modification. Metalloproteins containing Fe-S clusters (Fe-S proteins) can be identified in organisms from all kingdoms of life [1,4,5]. In Fe-S proteins, the formation of the clusters is often spontaneous *in vitro*, when the apo-protein is exposed to inorganic iron and sulfur sources [6], but requires dedicated biogenesis pathways *in vivo* [7–10]. The presence of Fe-S cluster is a major determinant of correct protein folding [11].

The oxygenation of the Earth's atmosphere by photosynthetic organisms created multiple challenges to Fe-S proteins. By oxidizing environmental iron to the iron(III) state, which rapidly precipitates as ferric hydroxide or forms insoluble complexes with anions, dioxygen reduced the bioavailability of this essential metal drastically. Thus as dioxygen accumulated in the atmosphere, iron became a limiting nutrient in many aerobic habitats. Furthermore, reactive oxygen species (ROS) are able to convert exposed Fe-S clusters to unstable inorganic species that quickly decompose [12,13]. Indeed, the integrity of Fe_4_S_4_ clusters in proteins such as aconitase and succinate dehydrogenase is frequently used to evaluate oxidative stress [14]. Consequently, aerobic organisms had to tackle the above challenges throughout their evolution. This entailed the development of specialized systems for iron uptake (in order to compensate the low bioavailability of the metal) [15,16] and Fe-S biogenesis [17], and of suitable scavenging and defense systems against ROS [18–20].

In this work, we compared the usage of Fe-S proteins in prokaryotic organisms with different life styles, and in particular with different dioxygen requirements. To this aim, we exploited the extensive amount of information made available by genome sequencing projects to predict the occurrence of Fe-S proteins in more than 400 organisms, based on protocols that we previously developed for the bioinformatics investigation of metalloproteins across the kingdoms of life [5,21]. As a result, we obtained a global view of the distribution of Fe-S proteins highlighting the specialization that occurred in different groups of prokaryotes as a function of their relationship with environmental dioxygen levels.

## Results

### Anaerobes have a higher content and a higher variety of iron sulfur proteins than aerobes

We analyzed the iron-sulfur genome of 434 prokaryotes (18 obligate aerobes, 29 obligate anaerobes, 214 aerobes, 130 aerotolerant anaerobes and 43 facultative anaerobes, S1 Table).

**Fig 1:**
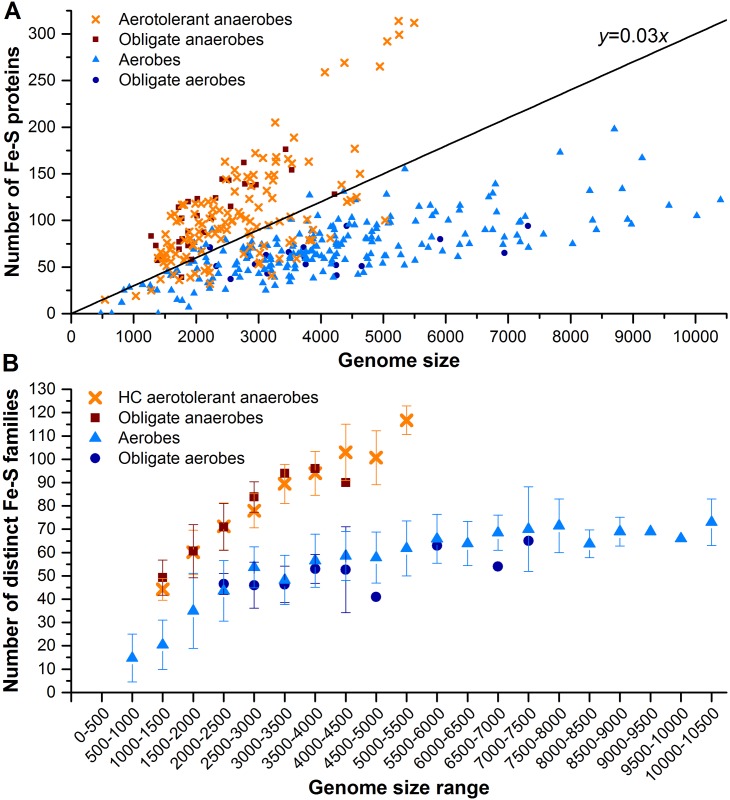
Dependence of the number of Fe-S proteins and Fe-S families on the genome size of the organisms. **A)** Number of putative Fe-S proteins as a function of the genome size in aerotolerant anaerobes (orange crosses), obligate anaerobes (red squares), aerobes (light blue triangles) and obligate aerobes (royal blue circles). The black line represent the threshold (3% of the genome content) used to separate LC aerotolerant anaerobes from HC aerotolerant anaerobes. **B)** Average number of distinct Fe-S families as a function of the genome size in HC aerotolerant anaerobes (orange crosses), obligate anaerobes (red squares), aerobes (light blue triangles) and obligate aerobes (royal blue circles).

The number of Fe-S proteins encoded by the genome of an organism depends on its genome size and its dioxygen requirements (Fig 1A). Such number increases proportionally to the genome size, but the fraction of Fe-S proteins is lower for the genomes of aerobic than anaerobic bacteria. In particular, obligate aerobes and aerobes (grouped together and named aerobes, hereafter) have a percentage of Fe-S proteins per genome below 3%. On the other hand, obligate anaerobes and the majority of aerotolerant anaerobes have more than 3% of Fe-S proteins in their genomes (S2 Table). For the subsequent analyses we grouped the organisms with more than 3% of Fe-S proteins and dubbed them high-content (HC) organisms. On average, aerobes and HC anaerobes have a fraction of Fe-S proteins in their genome of 1.8% ± 0.6% and 4.7% ± 1.0%, respectively (Table 1, last two lines). The genomes of only 28% of all aerotolerant anaerobes encode less than 3% of Fe-S proteins (low-content–LC—anaerobes, hereafter).

**Table 1:** Average percentage of Fe-S proteins encoded in the genome of each group of organisms, divided per dioxygen requirement. Columns 3–5 report the total number of organisms and the number of eubacterial and archaeal organisms of each type analyzed.

Dioxygen requirement	Average % of Fe-S proteins	# organisms	# eubacteria	# archaea
Obligate aerobes	1.7 ± 0.5	18	17	1
Aerobes	1.8 ± 0.7	214	196	18
Facultative anaerobes	2.3 ± 0.6	43	43	0
Aerotolerant anaerobes	4.0 ± 1.4	130	102	28
*LC aerotolerant anaerobes*	*2*.*2 ± 0*.*4*	*36*	*34*	*2*
*HC aerotolerant anaerobes*	*4*.*6 ± 1*.*0*	*94*	*68*	*26*
Obligate anaerobes	4.8 ± 1.0	29	17	12
All aerobic organisms	1.8 ± 0.6	232	213	19
All HC anaerobic organisms	4.7 ± 1.0	123	85	38

To analyze Fe-S families (i.e. families of homologous Fe-S proteins), we assigned each Fe-S protein to a precompiled COG (Cluster of Orthologous Groups) by exploiting the COG database [22]. Obligate aerobes and aerobes share the same set of families (S1A Fig); the same holds for HC anaerobes (S1B Fig). This observation supports our choice of considering aerobes and HC anaerobes as two independent groups.

For essentially all genome sizes, the number of distinct Fe-S families in HC anaerobes is higher than in aerobes. This difference increases with increasing genome size. Indeed, organisms having about 2,250 genes in their genome contain on average 44 ± 12 or 71 ± 10 Fe-S families depending on whether they are aerobic or not, yielding a difference of about 27 families (Fig 1B). For genomes of about 5,250 genes the difference increases to about 55 families.

For aerobic organisms, the number of distinct Fe-S families levels off at about 65 families for genomes with at least 4,000 genes (Fig 1B). Thus, the higher number of Fe-S proteins in the aerobes with larger genomes is not due to a higher number of different Fe-S families, but to a higher number of co-orthologous proteins (i.e., paralogs that were duplicated after speciation). Such proteins belong to the same Fe-S family and have a different specialization (e.g. enzymes performing the same catalysis but on different substrates) [23].

### HC anaerobes and aerobes share a common core of Fe-S families, which mostly bind Fe_4_S_4_ clusters

**Fig 2:**
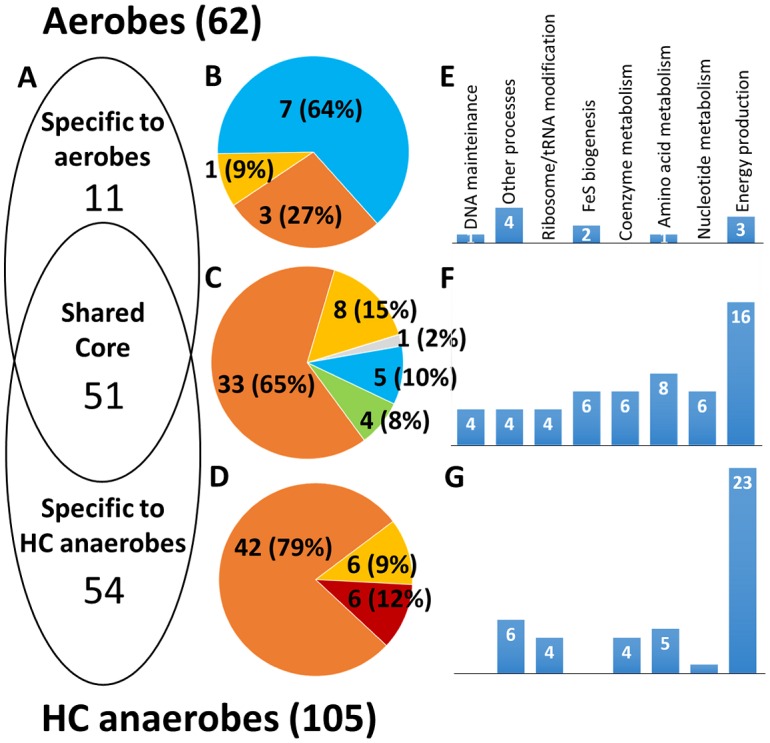
Frequently occurring (i.e. present in at least 30% organisms) families of Fe-S proteins in aerobes and HC anaerobes. (A): Venn diagram showing the distribution of the 116 frequently occurring Fe-S families within aerobes and HC anaerobes. (B), (C), and (D): Pie charts showing the types of Fe-S cluster (blue: Fe_2_S_2_; orange: Fe_4_S_4_; green: Fe_3_S_4_; yellow: two or more of Fe_2_S_2_-Fe_4_S_4_-Fe_3_S_4_; red: FeCys_4_; grey: unknown type) associated with families conserved in aerobes (B), in anaerobes (D), and in both (C). (E), (F), and (G): histograms showing the number of families associated with specific functional processes in aerobes (E), in anaerobes (G), and in both (F). More than one functional process may be associated with a family. Unknown functional processes are excluded from the count.

**Fig 3:**
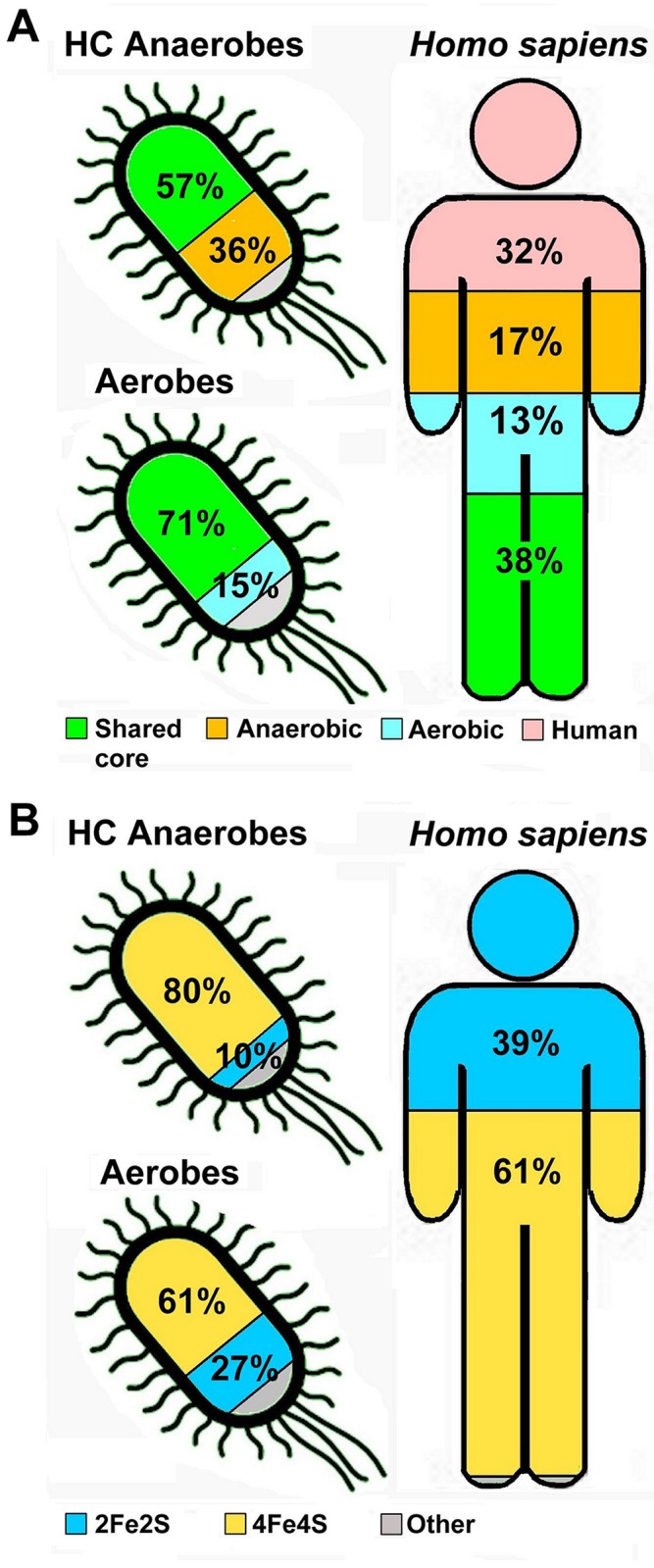
The occurrence of (A) and the Fe-S cluster type (B) in the Fe-S families of humans, aerobes and HC anaerobes. In panel A, human Fe-S families that did not map to any prokaryotic family of Fig 2 are classified as “Human”. Panel B displays the average fraction of proteins binding at least one cluster of the Fe_2_S_2_ or of the Fe_4_S_4_ type.

Aerobes and HC anaerobes encode, respectively, 62 and 105 frequently occurring Fe-S families, i.e. families that are present in at least 30% of the organisms (S3 Table). 51 families are common to both groups of organisms (Fig 2A). This shared core accounts for 57% ± 9% of the Fe-S genomes in HC anaerobes and for 71% ± 8% of the Fe-S genomes in aerobes (Fig 3A).

The most common functional process associated with the Fe-S families in the shared core is *energy production and conversion* (16 families, Fig 2F). Proteins in these families are involved in processes that use the same enzymatic components in both aerobes and HC anaerobes (e.g. subunits of the NADH:ubiquinone oxidoreductase), or are related enzymes that have been recruited in different but functionally similar roles. For example, fumarate reductase and succinate dehydrogenase (COG0479, S3 Table) are enzymes involved in anaerobic and aerobic respiration, respectively, catalyzing the same reaction but in opposite directions. *Amino acid metabolism* is the second most common process in the shared core (8 families), followed by *nucleotide and coenzyme metabolism* and *Fe-S biogenesis*, each associated with 6 families (Fig 2F). All these families have similar roles in aerobes and anaerobes. On average, the Fe-S families of the shared core contain more proteins in HC anaerobes than in aerobes (S2 Fig). More than half of the families that are larger in HC anaerobes are involved in *energy production and conversion* (8 out of 15).

The majority of the Fe-S families included in the shared core bind a Fe_4_S_4_-type cluster (65%, Fig 2C). An additional 15% bind more than one type of cluster, but at least one of these is of the Fe_4_S_4_ type. Therefore, the fraction of common families that bind at least one Fe_4_S_4_ cluster is 80%.

### HC anaerobes are enriched in Fe_4_S_4_-binding proteins whereas aerobes are enriched in Fe_2_S_2_-binding proteins

HC anaerobes have 54 specific Fe-S families (i.e., Fe-S families encoded in at least 30% of the HC anaerobes and found in less than 30% of aerobes) whereas only 11 Fe-S families are specific to aerobes (Fig 2A, S3 Table). Specific Fe-S families account for 36% ± 9% of the Fe-S genomes in HC anaerobes and for 15% ± 7% of the Fe-S genomes in aerobes (Fig 3A).

E*nergy production and conversion* is the functional process that is most often associated with both the Fe-S families specific to HC anaerobes and those specific to aerobes. However, the number of families involved in this process is very different in the two cases, being 23 for HC anaerobes (and thus higher than the corresponding number of families in the shared core, i.e. 16) and only 3 for aerobes (Fig 2E–2G). *Amino acid metabolism* is the only other process associated with both aerobe-specific and HC anaerobe-specific Fe-S families. Instead, *Fe-S biogenesis* and *DNA maintenance* are associated only with aerobe-specific families, whereas *ribosome/tRNA modification*, *coenzyme metabolism* and *nucleotide metabolism* are associated only with HC anaerobe-specific families (Fig 2E and 2G).

Seven of the 11 Fe-S families specific to aerobes bind only Fe_2_S_2_ clusters (64%, Fig 2B), whereas none of those specific to HC anaerobes binds only this type of Fe-S cluster (Fig 2D). On the other hand, 42 of the 54 Fe-S families specific to HC anaerobes bind only Fe_4_S_4_ clusters, whereas 6 have a simple FeCys_4_ site. Because of this differential enrichment in Fe-S families binding different types of clusters, the Fe-S genomes of HC anaerobes have an 8:1 ratio between Fe_4_S_4_ and Fe_2_S_2_ clusters, whereas this ratio for aerobes is only slightly above 2:1 (Fig 3B).

The occurrence of Fe-S families specific to aerobes as well as to HC anaerobes raises the question as to whether Fe-S proteins found in only one of the two groups have homologs in the other group that, however, do not bind Fe-S clusters. For 37 of the 54 HC anaerobe-specific Fe-S families, homologs are present in only a negligible fraction of aerobes (S3 Fig). In the remaining Fe-S families, homologs are present in aerobes but they do not bind Fe-S clusters because they do not harbor the Fe-S-binding domain. Tryptophanyl-tRNA synthetase (i.e. COG0180) is the only case where the Fe-S-binding domain is still present in aerobes but it lacks the amino acidic pattern to bind the Fe-S cluster [24]. Similarly, the large majority of the 11 aerobic-specific Fe-S families do not have homologs in HC anaerobic organisms (S4 Fig).

### Facultative anaerobes are aerobic organisms with an additional set of anaerobe-specific energy-related Fe-S families whereas LC anaerobes harbor a simplified set of the Fe-S portfolio of HC anaerobes

**Fig 4:**
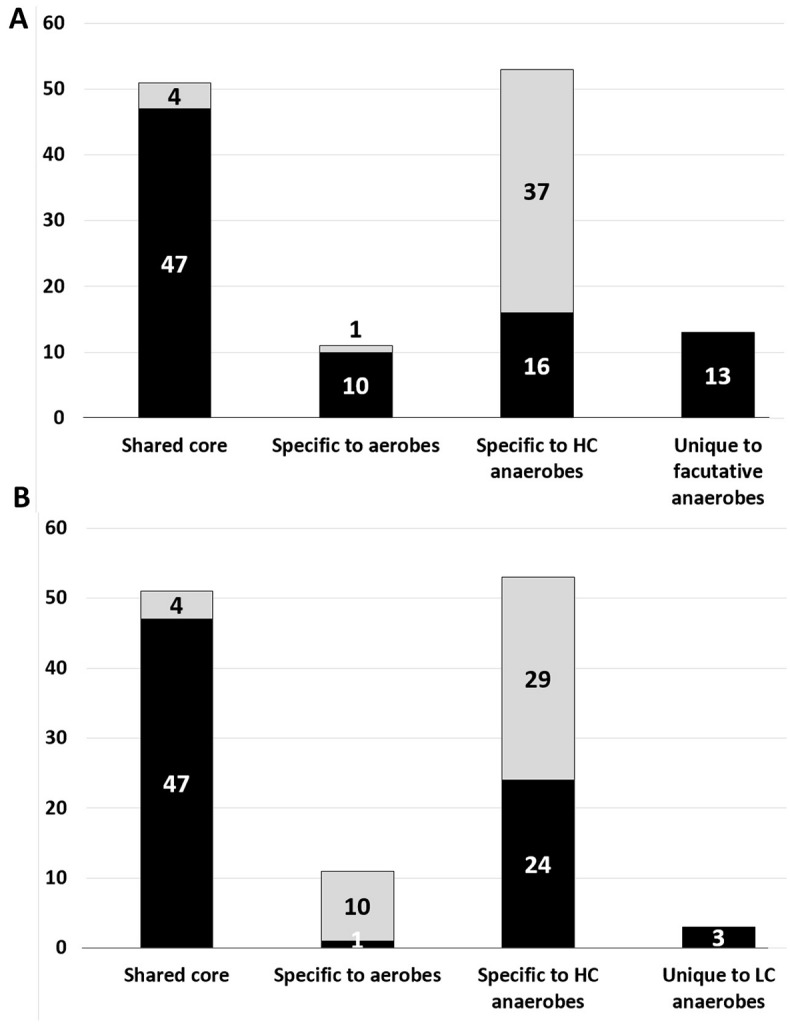
Distribution of the frequently occurring Fe-S families of aerobes and HC anaerobes within facultative anaerobes (A) and LC aerotolerant anaerobes (B). The black part of the column indicates the occurrence of the families of the three groups defined in Fig 2 within facultative anaerobes (A) and LC aerotolerant anaerobes (B). The grey part of the column indicates the absent families. The last columns correspond to Fe-S families unique to facultative anaerobes (A) and LC aerotolerant anaerobes (B), i.e. not mapping to any family in the Venn diagram of Fig 2.

Fe-S proteins represent 2.3% ± 0.6% of the genomes of facultative anaerobes (S1 Table). These organisms contain 47 out of the 51 (92%) Fe-S families constituting the shared core and 10 out of the 11 (91%) Fe-S families specific to aerobes (Fig 4A). In addition, facultative anaerobes contain 16 out of the 54 Fe-S families (30%) specific to HC anaerobes, and 13 specific Fe-S families. We can thus generally describe facultative anaerobes as having the same frequently occurring Fe-S families as aerobic organisms, plus an additional set of 29 families. More than half of this additional set (i.e. 18, 62%) is involved in *energy production and conversion*.

The same kind of analysis showed that LC aerotolerant anaerobes have a portfolio of 75 frequently occurring Fe-S families (Fig 4B) comprising most of the shared core (47 out of 51, 92%) and only a minority of Fe-S families from the aerobe- (1 out of 11, 9%) and HC anaerobe-specific (24 out of 54, 44%) groups. In addition, we detected 3 Fe-S families specific to LC anaerobes. Thus, LC anaerobes harbor a simplified version of the Fe-S portfolio of HC anaerobes, dominated by the Fe-S families of the shared core.

### Human cytosolic and nuclear Fe-S proteins have been inherited from anaerobes whereas mitochondrial proteins come from aerobes

The 70 known human genes encoding Fe-S proteins [25] are associated with 47 Fe-S families (S4 Table). Eighteen of these families (38%) are part of the shared core, 8 (17%) of the families specific to HC anaerobes, 6 (13%) of the families specific to aerobes, and 15 (32%) are rarely or never detected in prokaryotic organisms and thus can be regarded as specific to eukaryotes or to human (Fig 3A). Overall, human Fe-S proteins have a higher preference for Fe_2_S_2_ clusters than the average prokaryote (Fig 3B). In humans, Fe_2_S_2_ clusters are present in 39% of all Fe-S proteins. Instead, Fe_2_S_2_ clusters are the cofactors of 10% ± 5% Fe-S proteins from HC anaerobes and of 27% ± 9% Fe-S proteins from aerobes.

In the large majority of cases, human Fe-S families related to the shared core and to aerobe-specific families have mitochondrial localization for at least one co-ortholog. Instead, human Fe-S families related to HC anaerobe-specific families have nuclear and/or cytosolic localization, with only one exception. As described in the previous sections, the latter families have a strong preference for Fe_4_S_4_ clusters, whereas aerobe-specific families prefer Fe_2_S_2_ clusters (Fig 2). We previously showed that mitochondrial localization is nearly two-times more likely for a Fe_2_S_2_-binding than a Fe_4_S_4_-binding human Fe-S protein [25]. Instead, nuclear localization is significantly more likely for Fe_4_S_4_-binding proteins. Therefore, the homology relationships of human Fe-S proteins to their prokaryotic counterparts highlighted here provide a rationale for the observed distribution of Fe-S cluster types in the human cell.

At the functional level, mitochondrial Fe-S proteins that belong to the families present in prokaryotes are predominantly involved in *energy production and conversion* or in *Fe-S biogenesis*. On the other hand, the most common process for the families inherited from HC anaerobes is *ribosome/tRNA modification*. All the cellular compartments contain further Fe-S proteins that are specific to eukaryotes, involved in various functional processes [26].

### The chloroplast of *Arabidopsis thaliana* contains only one Fe-S protein inherited from anaerobes

We analyzed also the portfolio of Fe-S proteins in the chloroplast of Arabidopsis thaliana (Table 2). Among its 45 proteins there is a very similar occurrence of Fe_2_S_2_ and Fe_4_S_4_ clusters (41.7% versus 45.8%). This ratio (1:1.1) is much closer to that observed in the human mitochondrion (1:0.86) [25] than to the ratio in the human cytosol (1:1.8). It is also very distant from the ratios observed in prokaryotes (Fig 3B).

**Table 2:** The Fe-S proteins of the chloroplast of *Arabidopsis thaliana*.

**Cluster type**	**Number of proteins**	**Percentage over total**
Fe_2_S_2_	20	41.7
Fe_4_S_4_	22	45.8
Other	6	12.5
**Type of protein family**	**Number of proteins**	**Percentage over total**
Shared core	22	48.9
Aerobic-specific	13	28.9
Anaerobic-specific	1	2.2
*A*. *thaliana*-specific	9	20.0

In terms of their distribution with respect to the different groups of Fe-S protein families identified in Fig 2, the most striking observation is that the chloroplast is essentially devoid of anaerobic-specific proteins (1 protein only, corresponding to 2.2% of its entire content of Fe-S proteins). Nearly half of the proteins in the chloroplast belong to the shared core, whereas another 28.9% is from aerobic-specific families (Table 2). 20% of the Fe-S proteins in the chloroplast of *A*. *thaliana* do not have prokaryotic homologs. The near absence of Fe-S protein from families specific to HC anaerobes is a feature common to both the chloroplast and the mitochondrion.

## Discussion

The number of Fe-S proteins in the genome of prokaryotic organisms increases with increasing genome size, following an essentially linear relationship. The slope of this relationship depends strongly on the dioxygen requirement of the organisms under consideration. A 3% fraction of Fe-S proteins in the genome effectively discriminates the analyzed organisms into two major groups: all obligate anaerobes and the majority of aerotolerant anaerobes on one side (HC anaerobes) and all other organisms on the other side. The latter group includes all aerobes, facultative anaerobes and a minority of aerotolerant anaerobes that have a low content of Fe-S proteins.

We have identified a shared core of families of Fe-S proteins that occur frequently in all prokaryotes. Depending on their dioxygen requirement, the different sub-groups of organisms have additional specific Fe-S families, in a number that ranges from 11 for aerobes to 54 for HC anaerobes. Altogether, the families of the shared core and the families specific to the different groups represent 86–93% of the Fe-S genome of any prokaryote. Thus, only a relatively small minority of Fe-S proteins have a scattered occurrence among organisms with the same dioxygen requirements. This could be the result of the adaption to a particular environmental niche of each organism.

Aerobic organisms have a smaller fraction of Fe-S proteins in their genome than most of anaerobes. This is due to the decrease of the number of Fe-S families in aerobes as well as to the larger number of the co-orthologs in the families of the shared core in anaerobes than aerobes. The different size of the Fe-S portfolio in the various types of organisms does not affect all functional processes equally. For example, all organisms have a similar number of Fe-S proteins involved in coenzyme metabolism, which are part of the shared core. Instead, there is a significant difference for the Fe-S families involved in energy-related processes, in which the majority of the Fe-S proteins are involved. On average, HC anaerobes have more than twice Fe-S proteins involved in these processes than aerobes. Interestingly, the families involved in *energy production and conversion* in HC anaerobes comprise more specific Fe-S families than families of the shared core.

The diversification of Fe-S families involved in energy-related processes in HC anaerobes can be partly the result of different branching of the respiratory chains to adapt to the chemical properties of the final electron acceptor(s) of each anaerobic chain. On the contrary, all the respiratory processes of aerobes end up with dioxygen, thus reducing the need for multiple specialized branches. In line with this, facultative anaerobes, i.e. aerobic organisms that can survive in anaerobic environments, can be classified as aerobic organisms additionally endowed with Fe-S families common to anaerobes or with their own specific families. The majority of these additional families are involved in energy-related processes.

Aerobes also have their specific Fe-S families, involved in various functional processes (Fig 2). An intriguing feature of these families is that in the majority of cases they bind Fe_2_S_2_ clusters, which are instead a small fraction in the shared core families as well as in the Fe-S families specific to other types of organisms. Thus, in aerobes there is a sizeable reduction in Fe_4_S_4_-binding proteins and a partial enrichment in Fe_2_S_2_-binding proteins. This could be due to the lower bioavailability of iron for aerobic organisms. Furthermore, aerobes have to deal with reactive oxygen species (ROS), produced as byproducts of normal metabolism, such as superoxide. Superoxide is more damaging to Fe_4_S_4_ than Fe_2_S_2_ clusters. Indeed, it promotes the release of ferrous ions from Fe_4_S_4_ cofactors endorsing the activation of related regulatory mechanisms [27–29]. As an evolutionary response, it is thus plausible that aerobic organisms have favored the use of Fe_2_S_2_ with respect to that of Fe_4_S_4_. Note that ROS production is associated with aerobic respiration, being linked to dioxygen reduction, thus justifying the preferential use of Fe_2_S_2_ clusters by mitochondrial Fe-S proteins [30].

By analyzing human Fe-S proteins in the light of the present approach, it appears that Fe-S families in mitochondria and chloroplasts are directly related to prokaryotic proteins of the shared core and of aerobic families, whereas nuclear and cytoplasmic Fe-S proteins are related to anaerobic families. These observations are consistent with the mitochondrial endosymbiont being an aerobic alpha-proteobacterium [31] as well as with the cyanobacterial origin of chloroplasts [32]. Furthermore, there has been evolution of new proteins (predominantly binding Fe_2_S_2_ clusters) that are present in all organelles at similar extent. The present findings are in general agreement with the model for the origin of eukaryotic cells where the endosymbiosis event giving rise to mitochondria occurred after development of some intracellular structures, such as the early nucleus [33][34]. Indeed, we identified homologs of archaeal anaerobic Fe-S proteins both in the cytoplasm and in the nucleus, but only one with mitochondrial localization. It is likely that the mitochondrial endosymbiont brought the ISC machinery to the eukaryotes [35–37]. The ISC machinery matures nearly all human Fe-S proteins of the shared core as well as of families specific to aerobes. The CIA machinery, which is considered a eukaryotic-specific innovation [38,39], performs the maturation of the Fe-S proteins inherited from anaerobes.

The overall evolutionary landscape of Fe-S families here presented is consistent with an ancient core of mainly Fe_4_S_4_-binding proteins, which has been trimmed down because of the transition from a reducing to an oxidizing atmosphere. At the same time, the diversification within the families of the shared core proteins has been modest for organisms exposed to dioxygen. Also some aerotolerant anaerobes experienced a shrinkage of their Fe-S portfolio with respect to the ancient core, as evidenced by their lower number of Fe-S proteins with respect to obligate anaerobes. The occurrence in the shared core of one or more Fe-S families for a given functional process likely depends on how early each family appeared in the course of evolution and on the sensitivity of its Fe-S clusters to dioxygen. The latter aspect should be largely affected by serendipity for the most ancient families that developed in the primordial reducing atmosphere where the chemical threat of dioxygen was absent. This makes it difficult to hypothesize a rationale for the distribution of the various functional process of Fig 2, based only on the data currently available.

In general, it appears that evolution selected a differently sized portfolio of Fe-S-dependent functions in aerobes and anaerobes by recruiting entirely different protein families. When new Fe-S families evolved within aerobes, they favored the use of the more stable, less iron demanding, Fe_2_S_2_ cluster. Less commonly, evolution converted a family of Fe-S proteins into Fe-S-independent proteins, e.g. by removing the protein domain responsible for the binding of the inorganic cofactor.

## Materials and Methods

Using the approach described in [21] as implemented in the RDGB program [40], we predicted the iron-sulfur proteins encoded by the genomes of 434 prokaryotes (59 Archaea, 375 Bacteria, see Table 1) selected to be representative of the various branches of the tree of life (S1 Table). Our search started from 102 Pfam profiles: 80 with an associated Fe-S-binding 3D structure and 22 simply annotated as Fe-S-binding domains. The 3D structure of a metalloprotein can be used to define both the type of Fe-S cluster bound by the domain and the pattern of amino acids that are involved in the interaction of the protein with the metal cofactor. Such a pattern, which is called the metal binding pattern (MBP) [41], is a regular expression defining the identity and spacing of the metal-binding residues, for example, CX(4)CX(20)H, where X is any amino acid. For the profiles with an associated 3D structure, the MBP can be used as a filter to remove false positives [21] and the corresponding Fe-S cluster type is assigned as the potential cofactor. We matched the profiles mentioned above to 1131 3D structures of iron–sulfur proteins available from the Protein Data Bank, corresponding to 293 distinct MBPs.

We classified all organisms in five types based on their dioxygen requirement, i.e. obligate aerobes, aerobes, facultative anaerobes, aerotolerant anaerobes and obligate anaerobes. Information on the dioxygen requirement of the various organisms analyzed was retrieved from the Genomes OnLine Database [42]. We divided aerotolerant anaerobes into two subgroups depending on whether the fraction of Fe-S proteins in their genome was lower than 3% (low content, LC) or not (high content, HC).

We mapped each predicted Fe-S protein to the COG database [22] in order to assign it to a protein family (Fe-S family). Each COG was then assigned to one or more of the 17 functional processes used to classify the COG dataset [22]. Fe-S families encoded in at least 30% of the organisms of each type were dubbed “frequently occurring” and constituted the focus of most of our analyses. We manually checked the annotation of the functional processes of all frequently occurring Fe-S families.

We put all human Fe-S proteins that we addressed in a previous work [25] in relationship to the COGs identified as described in previous paragraph. In particular, we mapped each human Fe-S protein to aerobes or HC anaerobes based on the fraction of organisms containing at least one homolog to that protein.

## Supporting Information

S1 Fig (A) Percentage of aerobic organisms that encode a given Fe-S family (y axis) as a function of the percentage of obligate aerobic organisms that encode the same family (x axis). (B) Percentage of aerotolerant HC organisms that encode a given Fe-S family (y axis) as a function of the percentage of obligate anaerobic organisms that encode the same family (x axis).
(TIF)

S2 Fig Average number of co-orthologs found in Fe-S families of the shared core. Blue columns are for aerobes; red columns are for and anaerobes.
(TIF)

S3 Fig Occurrence in aerobes of the Fe-S families specific to HC anaerobes. For each Fe-S family specific of HC anaerobes (i.e. conserved in at least 30% the HC anaerobes and in less than 30% aerobes) the graph shows the percentage of aerobic organisms which (i) do not have a corresponding family member (grey); (ii) have members without a Fe-S-binding domain (green); (iii) have members with a Fe-S-binding domain but without the Fe-S-binding site (blue) and (iv) have members with both the Fe-S-binding domain and the Fe-S-binding site, and thus binds Fe-S (yellow). The corresponding percentage of HC anaerobes which contain Fe-S binding families are also indicated (black crosses).
(TIF)

S4 Fig Occurrence in HC anaerobes of the Fe-S families specific to aerobes. For each Fe-S family specific of aerobes (i.e. conserved in at least 30% aerobes and in less than 30% HC anaerobes) is reported the percentage of HC anaerobic organisms which (i) do not have a corresponding family member (grey); (ii) have members without a Fe-S-binding domain (green); (iii) have members with a Fe-S-binding domain but without the Fe-S-binding site (blue) and (iv) have members with both the Fe-S-binding domain and the Fe-S-binding site, and thus binds Fe-S (yellow). The corresponding percentage of aerobes which contain Fe-S binding families are also indicated (black crosses).
(TIF)

S1 Table Fe-S proteins predicted in obligate aerobes, aerobes, facultative anaerobes, aerotolerant anaerobes and obligate anaerobes. (XLSX)

S2 Table Number of Fe-S proteins and Fe-S families per each organism analyzed (obligate aerobes, aerobes, facultative anaerobes, aerotolerant anaerobes and obligate anaerobes are grouped in separate tabs). (XLSX)

S3 Table Families (COGs) that correspond to the predicted Fe-S proteins and analysis of the frequently occurring Fe-S families in HC anaerobes and aerobes. (XLSX)

S4 Table Families (COGs) that correspond to the 70 predicted Fe-S proteins in humans and their mapping to families of aerobic and HC anaerobic organisms. (XLSX)
