# Supplementary figures and images for: The Relationship between Environmental Dioxygen and Iron-Sulfur Proteins Explored at the Genome Level
Source: PLoS One. 2017 Jan 30;12(1):e0171279. doi: 10.1371/journal.pone.0171279 (PMC5279795; doi:10.1371/journal.pone.0171279)

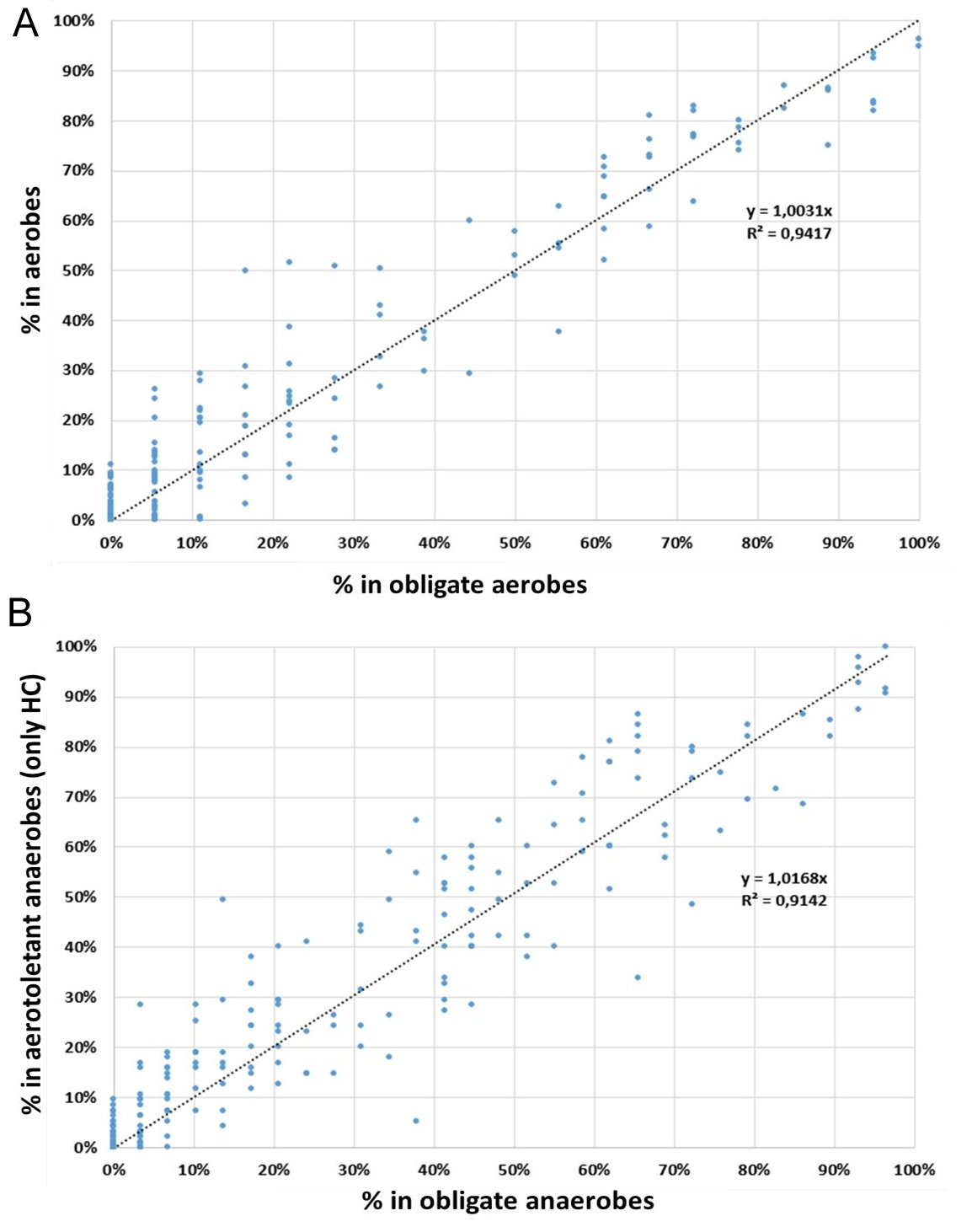

Supplement: S1 Fig — (A) Percentage of aerobic organisms that encode a given Fe-S family (y axis) as a function of the percentage of obligate aerobic organisms that encode the same family (x axis). (B) Percentage of aerotolerant HC organisms that encode a given Fe-S family (y axis) as a function of the percentage of obligate anaerobic organisms that encode the same family (x axis). (TIF) [file pone.0171279.s001.tif]

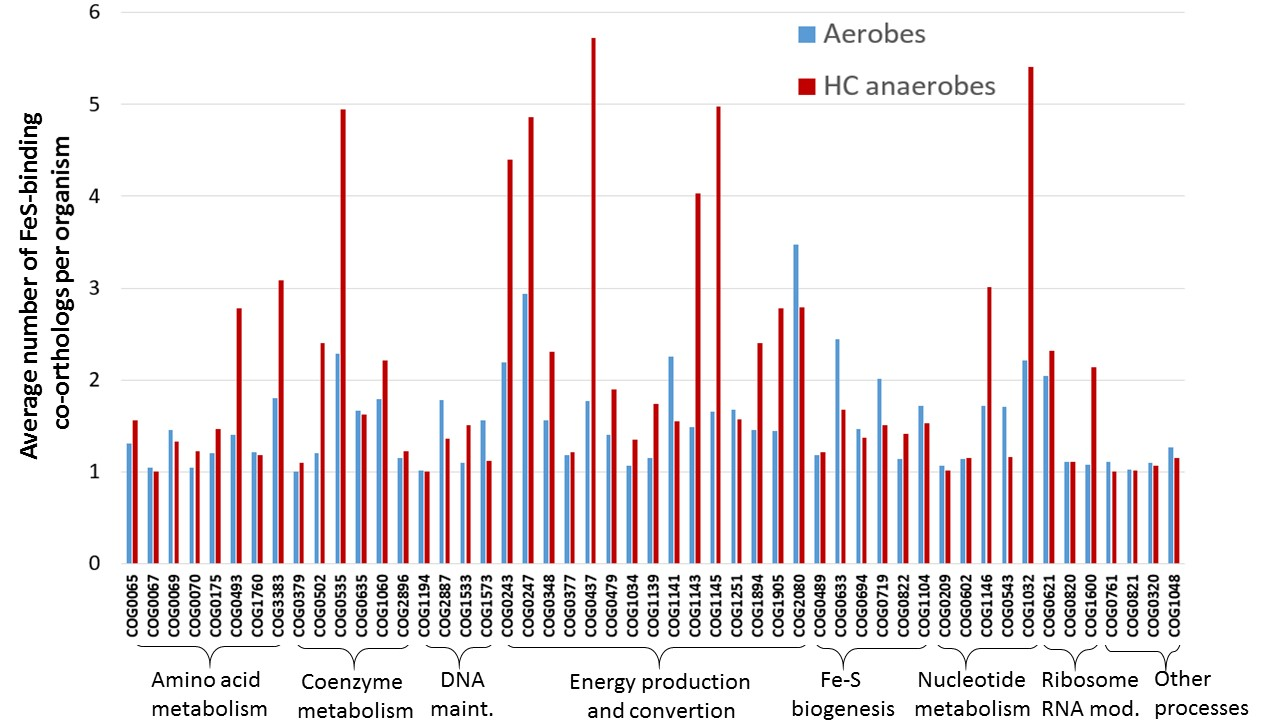

Supplement: S2 Fig — Blue columns are for aerobes; red columns are for and anaerobes. (TIF) [file pone.0171279.s002.tif]

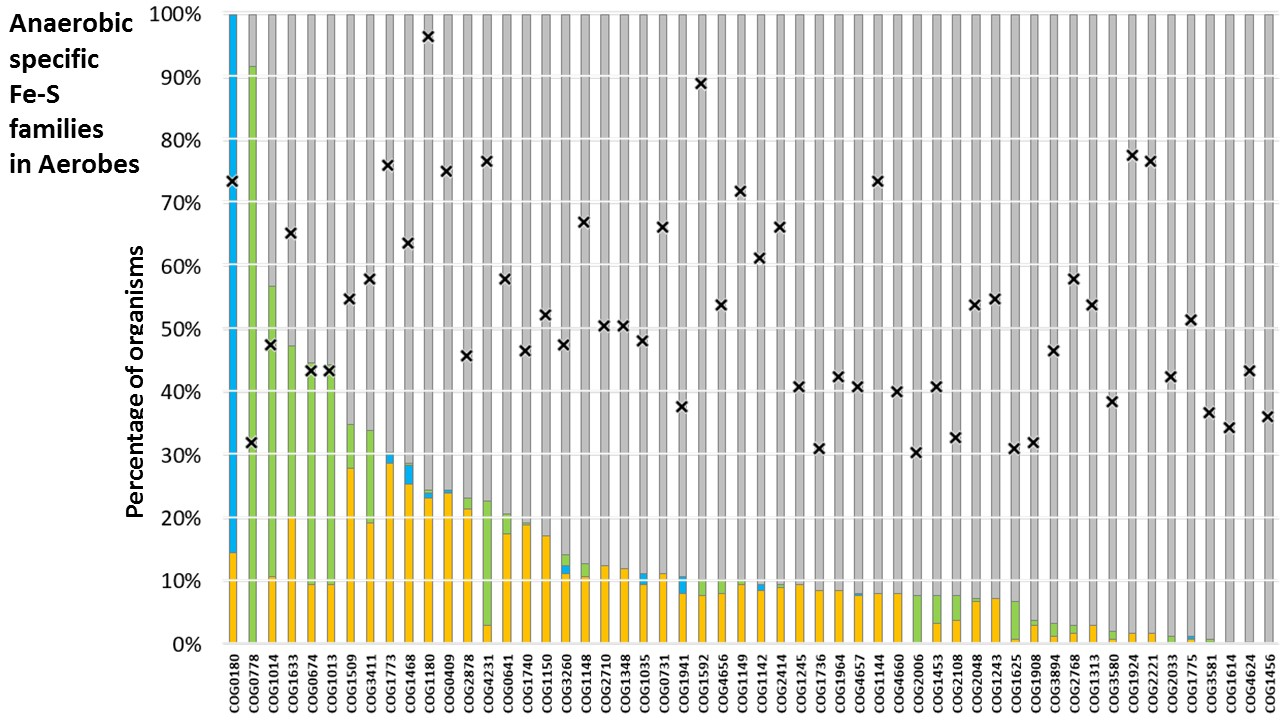

Supplement: S3 Fig — For each Fe-S family specific of HC anaerobes (i.e. conserved in at least 30% the HC anaerobes and in less than 30% aerobes) the graph shows the percentage of aerobic organisms which (i) do not have a corresponding family member (grey); (ii) have members without a Fe-S-binding domain (green); (iii) have members with a Fe-S-binding domain but without the Fe-S-binding site (blue) and (iv) have members with both the Fe-S-binding domain and the Fe-S-binding site, and thus binds Fe-S (yellow). The corresponding percentage of HC anaerobes which contain Fe-S binding families are also indicated (black crosses). (TIF) [file pone.0171279.s003.tif]

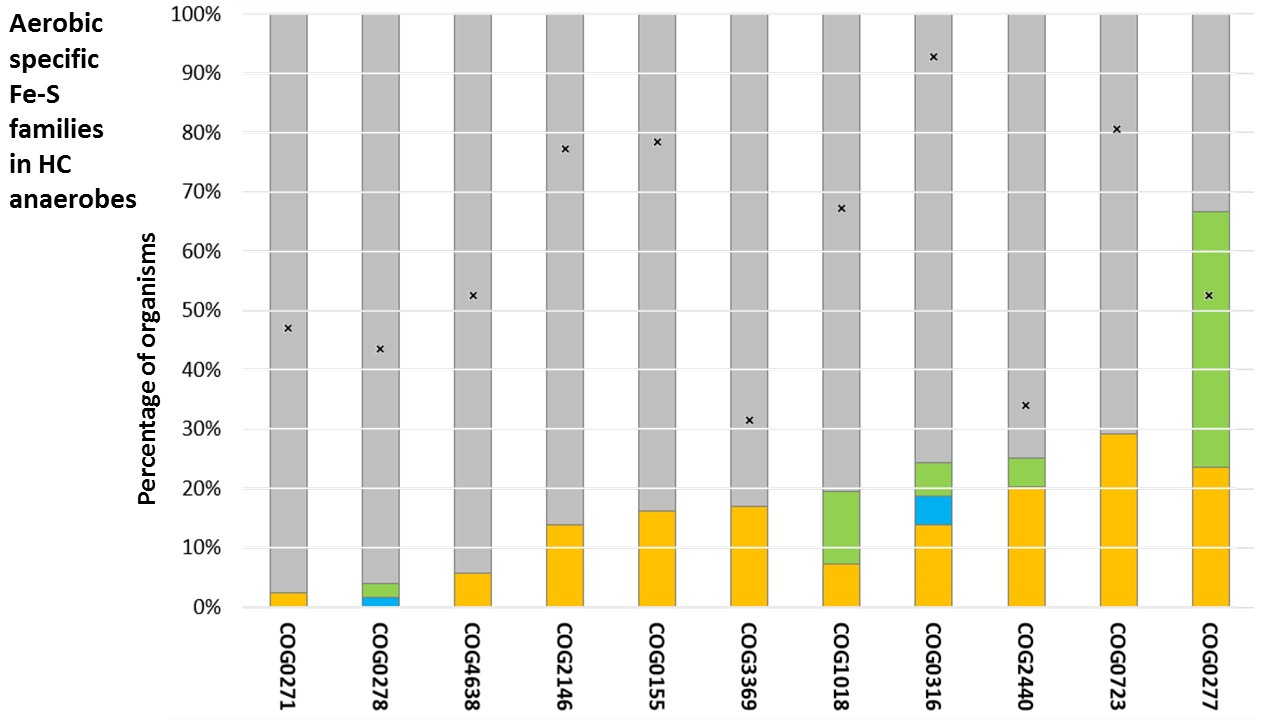

Supplement: S4 Fig — For each Fe-S family specific of aerobes (i.e. conserved in at least 30% aerobes and in less than 30% HC anaerobes) is reported the percentage of HC anaerobic organisms which (i) do not have a corresponding family member (grey); (ii) have members without a Fe-S-binding domain (green); (iii) have members with a Fe-S-binding domain but without the Fe-S-binding site (blue) and (iv) have members with both the Fe-S-binding domain and the Fe-S-binding site, and thus binds Fe-S (yellow). The corresponding percentage of aerobes which contain Fe-S binding families are also indicated (black crosses). (TIF) [file pone.0171279.s004.tif]
